# The latency pattern of Epstein–Barr virus infection and viral IL-10 expression in cutaneous natural killer/T-cell lymphomas

**DOI:** 10.1054/bjoc.2000.1687

**Published:** 2001-04

**Authors:** Z-G Xu, K Iwatsuki, N Oyama, M Ohtsuka, M Satoh, S Kikuchi, H Akiba, F Kaneko

**Affiliations:** Department of Dermatology, Fukushima Medical University School of Medicine, Hikarigaoka 1, Fukushima, 960-1295, Japan; Department of Dermatology, Graduate School of Medicine and Dentistry, Okayama University Graduate Schools, 2-5-1 Shikata-cho, Okayama, 700-8558, Japan

**Keywords:** Epstein–Barr virus, NK/T-cell lymphomas, latency, viral IL-10, bcl-2 homologue, LMP, EBNA

## Abstract

The nasal type, extranodal natural killer or T(NK/T)-cell lymphoma is usually associated with latent Epstein–Barr virus (EBV) infection. In order to elucidate the EBV gene expression patterns in vivo, we examined eight patients with cutaneous EBV-related NK/T-cell lymphomas, including six patients with a NK-cell phenotype and two patients with a T-cell phenotype. The implication of EBV in the skin lesions was determined by the presence of EBV-DNA, EBV-encoded nuclear RNA (EBER) and a clonality of EBV-DNA fragments containing the terminal repeats. Transcripts of EBV-encoded genes were screened by reverse transcription- polymerase chain reaction (RT-PCR), and confirmed by Southern blot hybridization. The expression of EBV-related antigens was examined by immunostaining using paraffin-embedded tissue sections and cell pellets of EBV-positive cell lines. Our study demonstrated that all samples from the patients contained EBV nuclear antigen (EBNA)-1 mRNA which was transcribed using the Q promoter, whereas both the Q promoter and another upstream promoter (Cp/Wp) were used in EBV-positive cell lines, B95.8, Raji and Jiyoye. Latent membrane protein-1 (LMP-1) mRNA was detected in seven of eight patients and all cell lines, whereas EBNA-2 transcripts were found only in the cell lines. Immunostaining showed no LMP-1, EBNA-2 or ZEBRA antigens in the paraffin-embedded tissue sections, although they were positive in the cell line cells. Latent BHRF1 transcripts encoding bcl-2 homologue and BCRF1 transcripts encoding viral interleukin (vIL)-10 were detected in one and two of eight patients, respectively. A patient with NK-cell lymphoma expressing both transcripts died of rapid progression of the illness. Our results indicate that the restricted expression of the latency-associated EBV genes and the production of vIL-10 and bcl-2 homologue may favour tumour growth, evading the host immune surveillance. © 2001 Cancer Research Campaign http://www.bjcancer.com


British Journal of Cancer (2001) 84(7), 920–925
© 2001 Cancer Research Campaign
doi: 10.1054/ bjoc.2000.1687, available online at http://www.idealibrary.com on IDEAL®

http://www.bjcancer.com

### The latency pattern of Epstein–Barr virus infection and viral IL-10 expression in cutaneous natural killer/T-cell lymphomas

**Z-G Xu[1], K Iwatsuki[2], N Oyama[1], M Ohtsuka[1], M Satoh[1], S Kikuchi[1], H Akiba[1] and F Kaneko[1]**

[1]Department of Dermatology, Fukushima Medical University School of Medicine, Hikarigaoka 1, Fukushima 960-1295, Japan
[2] Department of Dermatology, Graduate School of Medicine and Dentistry, Okayama University Graduate Schools, 2-5-1 Shikata-cho, Okayama 700-8558, Japan

**Summary** The nasal type, extranodal natural killer or T(NK/T)-cell lymphoma is usually associated with latent Epstein–Barr virus (EBV) infection. In order to elucidate the EBV gene expression patterns in vivo, we examined eight patients with cutaneous EBV-related NK/T-cell lymphomas, including six patients with a NK-cell phenotype and two patients with a T-cell phenotype. The implication of EBV in the skin lesions was determined by the presence of EBV-DNA, EBV-encoded nuclear RNA (EBER) and a clonality of EBV-DNA fragments containing the terminal repeats. Transcripts of EBV-encoded genes were screened by reverse transcription- polymerase chain reaction (RT-PCR), and confirmed by Southern blot hybridization. The expression of EBV-related antigens was examined by immunostaining using paraffin-embedded tissue sections and cell pellets of EBV-positive cell lines. Our study demonstrated that all samples from the patients contained EBV nuclear antigen (EBNA)-1 mRNA which was transcribed using the Q promoter, whereas both the Q promoter and another upstream promoter (Cp/Wp) were used in EBV-positive cell lines, B95.8, Raji and Jiyoye. Latent membrane protein-1 (LMP-1) mRNA was detected in seven of eight patients and all cell lines, whereas EBNA-2 transcripts were found only in the cell lines. Immunostaining showed no LMP-1, EBNA-2 or ZEBRA antigens in the paraffin-embedded tissue sections, although they were positive in the cell line cells. Latent BHRF1 transcripts encoding bcl-2 homologue and BCRF1 transcripts encoding viral interleukin (vIL)-10 were detected in one and two of eight patients, respectively. A patient with NK-cell lymphoma expressing both transcripts died of rapid progression of the illness. Our results indicate that the restricted expression of the latency-associated EBV genes and the production of vIL-10 and bcl-2 homologue may favour tumour growth, evading the host immune surveillance. © 2001 Cancer Research Campaign http://www.bjcancer.com

**Keywords**: Epstein–Barr virus; NK/T-cell lymphomas; latency; viral IL-10; bcl-2 homologue; LMP; EBNA

Several lines of evidence have shown the association of latent Epstein–Barr virus (EBV) infection with the nasal-type, extranodal natural killer or T (NK/T)-cell lymphoma which occurs predominantly in Asia and Mexico (Harabuchi et al, 1990; Su et al, 1991; Jaffe et al, 1996; Harris et al, 1999). This type of lymphoma primarily affects the nasal cavity and skin, exhibiting histopathological features of angiodestructive infiltration and tissue necrosis (Su and Hsieh, 1992; Jaffe et al, 1996; Chan et al, 1997; Iwatsuki et al, 1997), and a frequent complication of haemophagocytic syndrome (Jaffe et al, 1983, 1996). In addition to the presence of EBV-DNA, EBV-encoded nuclear RNA (EBER) is usually present in the neoplastic cells. The detection of these molecules, therefore, provides a diagnostic clue for this disorder. Although viral proteins such as latent membrane protein (LMP) and EBV nuclear antigen (EBNA) may have a crucial role for EBV-induced cell transformation (Wang et al, 1985; Rickinson et al, 1987), the in vivo expression pattern of the virus-related molecules has not yet been clarified in the nasal type, NK/T-cell lymphoma.

*Received 4 April 2000*
*Revised 6 December 2000*
*Accepted 7 December 2000*

*Correspondence to:* K Iwatsuki; Email: keijiiwa@fmu.ac.jp

EBV-infected cells and neoplasms generally show one of three different patterns of latency-associated viral gene expression: latency I, II and III (Rowe et al, 1987; Kerr et al, 1992). EBV-infected lymphoblastoid cell lines and lymphoproliferative diseases arising in immunocompromised hosts express all nine latency-associated viral proteins, including six EBNAs (1, 2, 3A, 3B, 3C and LP) and three LMPs (1, 2A and 2B) (Rowe et al, 1987; Young et al, 1989; Hamilton-Dutoit et al, 1993). This pattern of EBV gene expression is designated as latency III.

In latency II, as noted in EBV-associated Hodgkin's disease and nasopharyngeal carcinoma, the EBNA-1, LMP-1 and LMP-2 genes but not other EBNA genes are transcribed (Brooks et al, 1992; Deacon et al, 1993). In latency I, EBV-infected cells express only EBNA-1, which is essential for episomal replication. This pattern is found in Burkitt's lymphomas (Tao et al, 1998) and gastric carcinomas (Sugiura et al, 1996).

The differences in the three patterns of EBV gene transcription are determined by cell phenotypes and the usage of gene promoters. The EBNA genes are transcribed as a single polycistronic RNA molecule which is spliced to give rise to expression of the individual EBNA molecules. The transcription in latency III is initiated from a promoter, located within the BamHI regions C and W (the C/W promoter, Cp/Wp) and results in a Y/U/K-spliced EBNA1 mRNA, whereas transcription of only EBNA-1 in latency I is driven by a downstream promoter located in the BamHI F and Q regions (the Q promoter, Qp), generating a Q/U/K-spliced mRNA without other EBNA products (Sample et al, 1991; Schaefer et al, 1991).

In addition to the latency-associated genes, EBV encodes proteins that modulate the host's immune system and inhibit apoptosis. The BHRF1 gene has partial sequence homology to the human bcl-2 gene (Henderson et al, 1993) and the BCRF1 gene encodes viral interleukin (IL)-10 (Moore et al, 1990). Furthermore, the BARF1-encoded protein functions as a soluble receptor for colony-stimulating factor 1 which inhibits interferon-α secretion from mononuclear cells (Cohen et al, 1999). The expression of these molecules may be implicated in the outgrowth of EBV-infected cells.

In order to clarify the latency pattern and the expression of EBV-encoded immunoregulatory molecules, we examined the biopsy specimens from eight Japanese patients with EBV-related cutaneous NK/T-cell lymphomas. We investigated transcripts of latency-associated EBV genes, the BHRF1 (bcl-2 homologue) and the BCRF1 (viral IL-10) genes in tumour tissues by reverse-transcription polymerase chain reaction (RT-PCR) and Southern blot hybridization.

### PATIENTS, MATERIALS AND METHODS

### Patients

Eight patients with nasal-type, extranodal NK/T-cell lymphoma including six patients with a NK-cell phenotype and two with a T-cell phenotype were enrolled in the study. The diagnoses were confirmed by immunophenotyping of the infiltrating cells and the detection of EBV-DNA, EBV-encoded RNA (EBER) and a clonality of EBV-DNA fragment containing the terminal repeat as described below. The clinical manifestations of two patients (NK3 and T1) have been reported in detail elsewhere (Ohtsuka et al, 1999).

### Tumour samples and cell lines

Paraffin-embedded skin biopsy specimens from the eight patients were examined for immunophenotypes, EBER and EBV antigens, including LMP-1, EBNA2 and ZEBRA. DNA samples from frozen skin biopsies were used for clonality analyses of T-cell receptor gene rearrangement and EBV terminal repeats, and for the determination of EBV gene transcripts. Two established EBV-positive Burkitt's lymphoma cell lines, Raji and Jiyoye, and one EBV-transformed lymphoblastoid cell line, B95.8, served as positive controls for EBV gene transcription. An EBV-negative T-cell line, Molt, was used as a negative control for RT-PCR. All cell lines were cultured in RPMI 1640 supplemented with 10% fetal bovine serum, 1 mM glutamine, 100 U/ml$^{-1}$ penicillin and 100 U ml$^{-1}$ streptomycin.

### In situ hybridization for EBER

In situ hybridization was performed on paraffin-embedded sections. After deparaffinization and digestion with 0.8% pepsin (DAKO, Glostrup), the sections were pre-hybridized with salmon sperm DNA and then hybridized with fluorescein isothiocyanate-labelled oligonucleotide probe for EBER1 and 2 (Novocastra, Newcastle upon Tyne) at 37˚C overnight. After post-hybridization washing with a combination of 150 mmol l$^{-1}$ sodium chloride and 15 mmol l$^{-1}$ sodium citrate (pH 7.0) at 50˚C, the specimens were incubated with alkaline phosphatase-conjugated antibody to fluorescein isothiocyanate (Novocastra). Positive signals were visualized after the addition of 5-bromo-4-chloro-3-indolylphosphate and nitroblue tetrazolium.

### Clonality analysis of EBV terminal repeats

Frozen tissues were minced on ice in buffer containing 15 mM sodium chloride, 15 mM Tris-HCl and 1 mM EDTA, and digested at 37˚C overnight with 0.025% proteinase K. Following extraction with phenol/chloroform and ethanol precipitation, DNA was resuspended in 10 mM Tris-HCl, 1 mM EDTA. Five μg DNA was digested with BamHI and then separated on a 0.8% agarose gel. The DNA was transferred to a nylon membrane and then hybridized with a digoxigenin-labeled EBV terminal fragment probe, an Xho-1 1.9 kb fragment (Raab-Traub and Flynn, 1986).

### mRNA preparation and RT-PCR

mRNAs were extracted from frozen skin biopsy specimens or 10$^{6}$ control cells using a Micro-FastTrack™ 2.0 kit (Invitrogen,

**Table 1** Oligonucleotides used in RT-PCR for EBV transcripts

| cDNA | Oligos | Oligonucleotide sequence (5′-3′) | Coordinated in B95.8 | Annealing temperature | Reference |
|---|---|---|---|---|---|
| EBNA1 | Y3 | TGGCGTGTGACGTGGTGTAA | 48397–48416 | 60˚C | Brooks et al, 1992 |
| | Q | GTGCGCTACCGGATGGCG | 62440–62457 | 55˚C | |
| | K | CATTTCCAGGTCCTGTACCT | 107986–107967 | | |
| | probe | AGAGAGTAGTCTCAGGGCAT | 67544–67563 | | |
| EBNA2 | H1 | AAGCGCGGGTGCTTAGAAGG | 48478–48459 | 55˚C | Oudejans et al, 1995a |
| | Y2 | ATTAGAGACCACTTTGAGCC | 47902–47921 | | |
| | probe | CTGTCCCGTATACACAGGGC | 54425–54406 | | |
| LMP1 | primer | GTGACTGGACTGGAGGAGCC | 169341–169322 | 62˚C | Tao et al, 1998 |
| | primer | GAGGGAGTCATCGTGGTGGTG | 168718–168738 | | |
| | probe | AGCCCTCCTTGTCCTCTA | 179325–169308 | | |
| BHRF1 | Y2 | ATTAGAGACCACTTTGAGCC | 47902–47921 | 55˚C | Oudejans et al, 1995a |
| | H2 | GTCAAGGTTTCGTCTGTGTG | 53830–63849 | 53˚C | |
| | H3 | TTCTCTTGCTGCTAGCTCCA | 54480–54461 | | |
| | probe | CTGTCCCGTATACACAGGGC | 54425–54406 | | |
| BCRF1 | primer | CGAAGGTTAGTGGTCACTCT | 9681–9700 | 57˚C | Miyazaki et al, 1993 |
| | primer | CACCTGGCTTTAATTGTCATG | 10186–10166 | | |
| | probe | TACCTGGAGGAAGTCATGCC | 9921–9940 | | |

*© 2001 Cancer Research Campaign* 

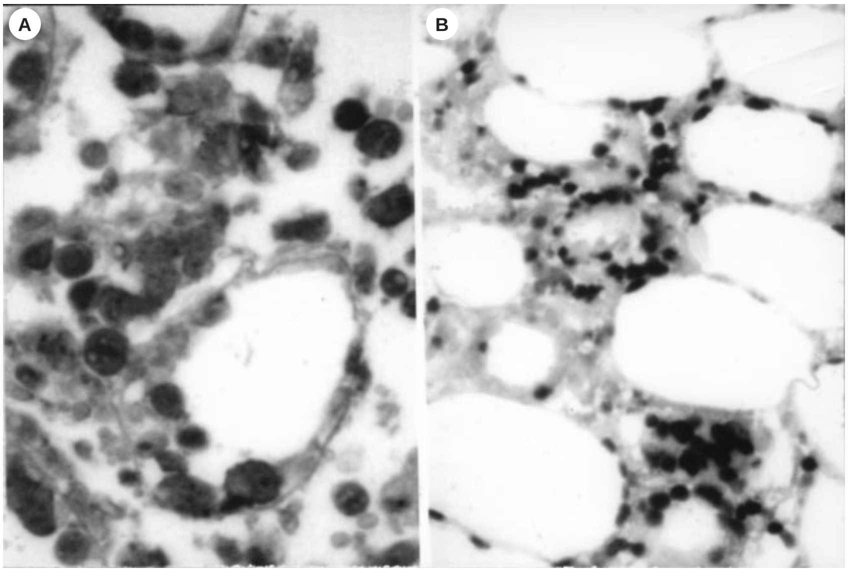

**Figure 1** Infiltrating cells in the skin lesions expressing (**A**) CD56 (× 600) and (**B**) EBER (× 400)

California). Reverse transcription was performed using a cDNA Cycle kit (Invitrogen). PCR amplification was done with an initial denaturation at 95˚C for 5 min, followed by 40 cycles consisting of 95˚C for 1 min, an optimal annealing temperature for 45 s, 72˚C for 1 min, and a final extension at 72˚C for 10 min. The RT-PCR products were transferred to nylon membranes, and then hybridized with biotin-labelled oligonucleotide probes as described previously (Xu et al, 2000). Transcription of EBV-encoded genes including the EBNA-1, EBNA-2, LMP-1, BHRF1 and BCRF1 genes was examined. The sequences of the primers, the annealing temperatures for PCR amplifications and the oligonucleotide probes for hybridization are described in Table 1.

### Immunostaining

In order to detect EBV antigens, monoclonal antibodies (DAKO) to LMP-1 (clone CS1-4), EBNA-2 (clone PE2) and ZEBRA (clone BZLF1) were used. Before incubation with anti-EBNA-2 or anti-ZEBRA antibody, deparaffinized tissue sections were boiled for 5 min in citrate buffer (0.1 mol $l^{-1}$, pH 6.0). Incubation with the antibodies was done at 4˚C overnight. For immunophenotyping of the infiltrating cells, monoclonal antibodies to CD2, 3, 4, 8, 16, 30, 45RO and 57 (DAKO) and CD56 (Novocastra) were used. The tissue sections were reacted with biotin-labeled anti-mouse immunoglobulins, followed by incubation with horseradish peroxidase-labelled streptavidin. Colorimetric reaction was performed by incubation with a substrate solution containing diaminobenzidine and hydrogen peroxide.

### RESULTS

### Clinicopathologic findings

The clinical features of six patients with a NK-cell phenotype (NK 1–6) and two patients with a T-cell phenotype (T1 and 2) are summarized in Table 2. Histologically, tissue necrosis and angiodestructive infiltrates were common in all patients at various degrees. All six patients with a NK-cell phenotype represented CD56 and cytoplasmic CD3 (CD3 ε) without any T-cell-specific antigens (Figure 1A) and positive signals for EBER (Figure 1B). $EBER^{+}$ cells ranged from 30–80% of the infiltrating cells. Southern blot hybridization demonstrated non-rearranged T-cell receptor genes (Figure 2). By contrast, rearranged bands of the T-cell receptor gene were detected in two patients (T1 and 2), although a few $CD56^{+}$ cells were observed in case T1 (data not shown). These findings confirmed that the tumour cells in cases T1 and T2 were of T-cell lineage.

DNA samples from five patients were subjected to clonality analyses of EBV. The results showed a single hybridization band

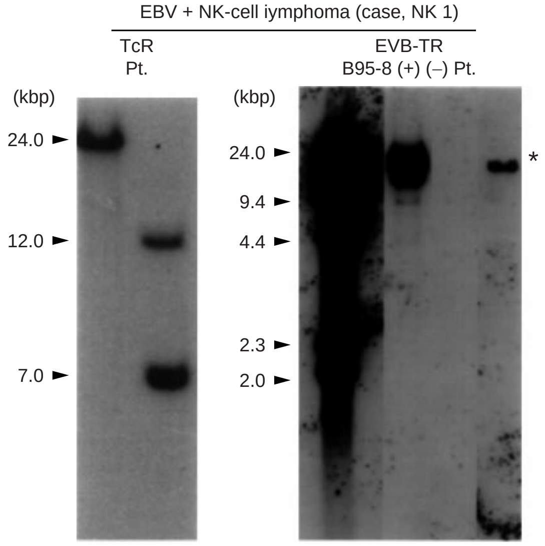

**Figure 2** Clonality analysis of a representative case of nasal-type, NK/T-cell lymphoma (NK-cell phenotype). Southern blot hybridization using a digoxigenin-labelled Xho-1 probe (Raab-Traub and Flynn, 1986) demonstrated a single band corresponding to EBV BamHI-digested fragments containing the same number of EBV terminal repeats (*right) and no rearrangement of TcR β gene (left). Pt = patient's sample, B95-8 = an EBV + cell line, (+) = EBV + lymphoma, (–) = EBV − lymphoma

© 2001 Cancer Research Campaign

**Table 2** Patients with cutaneous NK/T-cell lymphomas

| Case/age/sex | Involved organs | Immunophenotype | TCRβ | EBV-TR | Outcome |
|---|---|---|---|---|---|
| NK1/70/M | nose, skin | cCD3$^+$, CD56$^+$, 30$^-$, 45RO$^-$, 8$^{-/+}$, 4$^{-/+}$ | G | Clonal | Alive |
| NK2/63/F | nose, skin, LN | cCD3$^+$, CD56$^+$, 2$^+$, 57$^-$, 16$^-$, 30$^-$ | G | Clonal | Dead |
| NK3/16/M | skin | cCD3$^+$, CD56$^+$, 4$^-$, 8$^-$, 30$^-$ | G | ND | Dead |
| NK4/74/F | skin | cCD3$^+$, CD56$^+$, 30$^+$, 4$^-$, 8$^-$ | ND | Clonal | Alive |
| NK5/48/F | skin, LN | cCD3$^+$, CD56$^+$, 45RO$^+$, 8$^-$, 4$^-$ | G | Clonal | Alive |
| NK6/80/M | skin | cCD3$^+$, CD56$^+$, 4$^-$, 8$^-$, 30$^-$ | G | ND | Dead |
| T1/64/F | skin, LN | cCD3$^+$, CD56$^{-/+}$, 3$^{-/+}$, 4$^+$, 8$^+$, 45RO$^+$ | R | Clonal | Dead[a] |
| T2/36/F | skin | cCD3$^+$, CD56$^-$, 45RO$^+$ | R | ND | Dead |

NK = NK-cell lymphomas, T = T-cell lymphomas, LN = lymph node, TCR = T-cell receptor, EBV-TR = EBV terminal repeats, + = positive, G = germ, R = rearranged, ND = not done. [a]The cause of death was unrelated to lymphoma.

**Table 3** The expression patterns of EBV-encoded genes by NK/T-cell lymphomas

| Cases | EBER by ISH | EBV gene expression determined by RT-PCR | | | | | | | EBV antigen expression by immunostaining | | |
|---|---|---|---|---|---|---|---|---|---|---|---|
| | | EBNA1 | | EBNA2 | LMP-1 | BHRF1 | | BCRF1 | LMP-1 | EBNA | ZEBRA |
| | | YUK | QUK | | | Y2/H3 | H2/H3 | (vIL-10) | | | |
| NK1 | + | – | + | – | + | – | – | – | – | – | – |
| NK2 | + | – | + | – | + | + | – | + | – | – | – |
| NK3 | + | – | + | – | + | – | – | +/– | – | – | – |
| NK4 | + | – | + | – | + | – | – | – | – | – | – |
| NK5 | + | – | +/– | – | – | – | – | – | – | ND | ND |
| NK6 | + | – | + | – | + | – | – | – | ND | ND | ND |
| T1 | + | – | + | – | + | – | – | – | – | – | – |
| T2 | + | – | + | – | + | – | – | – | – | – | – |
| Molt | ND | – | – | – | – | – | – | – | – | – | – |
| B95.8 | + | + | + | + | + | + | + | + | + | + | + |
| Raji | ND | + | + | + | + | + | + | + | + | + | + |
| Jiyoye | ND | + | + | + | + | + | +/– | + | + | + | + |

EBER = EBV−encoded small nuclear RNA, ISH = in situ hybridization, + = positive; +/– = weakly positive, – = negative, ND = not done.

with a Xho-1 probe, indicating clonal proliferation of the neoplastic cells infected with EBV containing the same number of terminal repeats (Figure 2).

Four years after onset, four patients (NK2, 3, 6 and T2) had died because of progressive illness, and one patient (T1) died of a complication unrelated to lymphoma. Among them, a patient with NK-cell phenotype (NK2) had the most aggressive course, and died 6 months after onset.

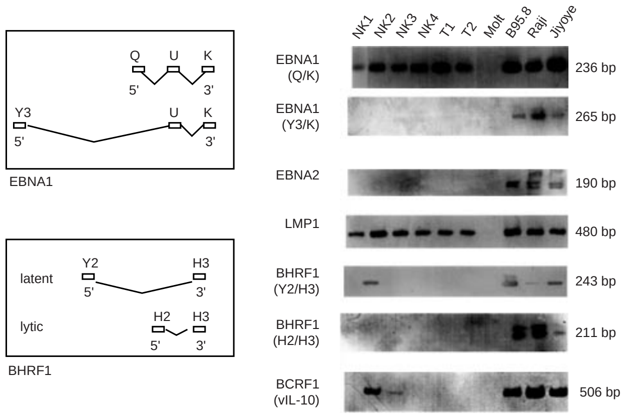

**Figure 3** Transcripts of EBV genes in NK/T-cell lymphoma. Schematic drawings indicate the complementary sites of the primer sets. The Q/U/K-spliced EBNA-1 and LMP-1 transcripts are detected in all EBV$^+$ NK/T-cell lymphomas, but neither Y3/U/K-spliced EBNA-1 nor EBNA-2 transcript is negative. Among the patients' samples, the latent Y2/H3(HF)-spliced BHRF1 transcript is positive in one case (NK2), and the BCRF1 transcript is present in two cases (NK2 and 3). NK = NK-cell lymphoma, T = T-cell lymphoma

### Expression pattern of latency-associated EBV genes

In order to determine the latency pattern of EBV infection in our patients, we analysed the transcripts of the EBNA-1, EBNA-2 and LMP-1 genes by RT-PCR. The EBNA-1 mRNA was found in all patients and EBV-positive cell lines such as B95.8, Raji and Jiyoye, whereas the EBNA-2 mRNA was absent in our patients but was present in the cell lines. The EBNA-1 promoters used to drive transcription were different between the patients and the cell lines. The Cp/Wp, which generates a Y/U/K-spliced form, was only used by cell lines (Table 3, Figure 3). By contrast, a downstream promoter, Qp, which produces a Q/U/K-initiated transcript, was used in both the cell lines and our patients. Therefore, the EBNA-1 transcript was driven from the Qp in NK/T-cell lymphomas without usage of the Cp/Wp. The LMP-1 mRNA was detected in seven of eight patients and the EBV-positive cell lines.

The expression of the BHRF1 gene, which has partial sequence homology to the human bcl-2 proto-oncogene, was studied using two different primer sets, Y2/H3 and H2/H3 for detection of possible latent (Y2/HF-spliced) and lytic (H2/HF-spliced) transcripts, respectively (Oudejans et al, 1995b). Both latent Y2/HF- and lytic H2/HF-spliced transcripts of the BHRF1 gene were detected in the EBV-positive cell lines, whereas only the latent

© 2001 Cancer Research Campaign 

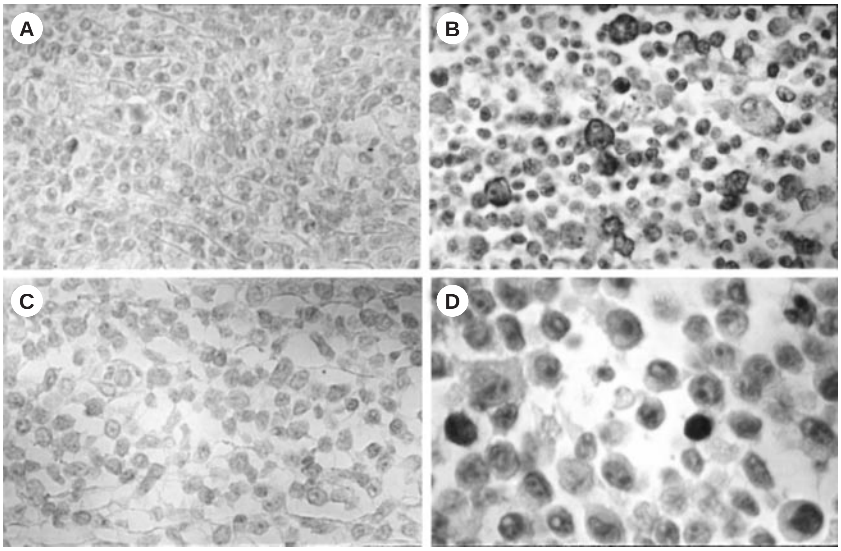

**Figure 4** Immunostaining of the LMP-1 and ZEBRA antigens in paraffin-embedded tissue and cell preparations from NK/T-cell lymphomas and an EBV+ lymphoblastic cell line, B95.8, respectively. Neither (**A**) LMP-1 nor (**C**) ZEBRA is present in the NK/T cell lymphoma, but both antigens (**B** and **D**, respectively) are positive in B95.8

Y2/HF-spliced transcript was detected in one (NK2) of eight patients with NK/T-cell lymphoma.

The BCRF1 gene, which encodes viral IL-10, was transcribed in all EBV-positive cell lines and two patients (NK2 and 3). The patient, NK2, who died of the most aggressive course of the illness, expressed both the BHRF1 (bcl-2 homologue) and BCRF1 (viral IL-10) transcripts.

### EBV antigen expression

The EBNA-2 and ZEBRA, an 'immediate-early' transcriptional activator was detected in the paraffin-embedded cell pellets of B95.8, Raji and Jiyoye cells, although the percentages of the positive cells varied in the cell lines (Figure 4). Neither EBNA-2- nor ZEBRA-positive cells were detected in the lesional tissue sections from our patients. With paraffin-embedded tissue sections, LMP-1 antigens were strongly expressed by the EBV-positive cell lines, but no LMP-1 antigens were detected in the biopsy specimens from the patients.

### DISCUSSION

Our study demonstrats that in addition to EBER, both the EBNA-1 and LMP-1 genes are transcribed in vivo in patients with EBV-associated NK/T-cell lymphomas, without any EBNA-2 gene transcript. This finding was further confirmed by the usage of promoters for the EBNA-1 gene in the patients: the Qp promoter was used in the NK/T-cell lymphomas, and both the Qp and Cp/Wp were used in the EBV-positive cell lines. When the Qp promoter is used, the Q/U/K-spliced EBNA-1 mRNA is generated, but the other latent genes, such as EBNA-2 and EBNA-3, cannot be transcribed because the Cp/Wp promoters are silent. These results indicate that the nasal-type, extranodal NK/T-cell lymphomas expressed viral antigens of EBNA-1 and LMP-1 in the latency II pattern. Unlike the latency III pattern observed in EBV-related lymphoproliferative disorders in patients with immunodeficiency (Hamilton-Dutoit et al, 1993), NK/T-cell lymphomas may minimize the expression of the latency-associated viral antigens which might be targeted by host immune system.

EBNA-1 is constantly expressed by EBV-infected cells in any latency pattern, but the host's immune response never occurs against the molecules (Levitskaya et al, 1995). LMP-1 acts as a direct oncogene to transform epithelial cells morphologically (Fahraeus et al, 1990) and prevent B-lymphoma cells from undergoing apoptosis by up-regulating the expression of cellular bcl-2 (Henderson et al, 1991). We could not detect LMP-1 antigens by immunostaining despite the presence of LMP-1 gene transcripts by RT-PCR. This discrepancy may be because RT-PCR is more sensitive than immunostaining using paraffin-embedded tissue sections.

The EBV BHRF1 gene encodes a protein which has sequence homology to cellular bcl-2, and this viral bcl-2 homologue protects cells from apoptosis (Henderson et al, 1993). High levels of the BHRF1 transcript are expressed in the lytic cycle, but differently spliced BHRF1 transcript was found during latency, although at much lower levels (Oudejans et al, 1995b). In our series, the latent (Y2/HF-spliced) transcript was present in one case (NK2), whereas no lytic (H2/HF-spliced) transcript was detected in the patients' samples. The BCRF1 gene encodes viral IL-10 which has more than 70% amino acid identity with human IL-10 (Moore et al, 1990). Human and viral IL-10 share several biological functions such as inhibition of interferon-$\gamma$ secretion and suppression of T-cell proliferation (Hsu et al, 1990). It has been postulated that the production of human IL-10 correlates with poor prognosis in non-Hodgkin's lymphoma (Blay et al, 1993). In our series, the BCRF1 gene was transcribed in two patients with cutaneous NK-cell lymphomas (NK2 and 3) who died after two years because of progression of the illness. In particular, a patient, NK2, with simultaneous expression of both viral IL-10 and viral bcl-2 homologue, did have an aggressive clinical course.

The BCRF1 gene encoding viral IL-10 was thought to be transcribed only during a lytic phase of EBV replication (Hudson et al, 1985), but was recently found to be expressed very early during B-cell infection (Miyazaki et al, 1993; Zeidler et al, 1997). Although the mechanism of the BCRF1 gene expression has been a controversial issue, we could not rule out the possibility that a small number of the neoplastic cells changed from the latent to the

 *© 2001 Cancer Research Campaign*

lytic cycle (Miller, 1990). Alternatively, bystander or reactive cells with lytic EBV infection might coexist in the lesions.

In conclusion, the latency II pattern of viral gene transcripts in the nasal-type, NK/T cell lymphomas (EBER$^+$, EBNA1$^+$, LMP-1$^+$, EBNA2$^-$) is distinct from that identified in B-cell system, and consistent with that identified in nasopharyngeal carcinomas. Furthermore, the expression of viral IL-10 and bcl-2 homologue might be responsible for tumour progression by interference with the host immune system and apoptosis, respectively.

### ACKNOWLEDGEMENTS

This study was supported by Grant-in-Aid for Scientific Research (C) (11670843) and Grant-in-Aid for Scientific Research on Priority Areas (C) (12218235) from the Ministry of Education, Science, Sports and Culture in Japan.

### REFERENCES

Blay JY, Burdin N, Rousset F, Lenoir G, Biron P, Philip T, Banchereau J and Favrot MC (1993) Serum interleukin-10 in non-Hodgkin's lymphoma: a prognostic factor. *Blood* **82**: 2169–2174

Brooks L, Yao QY, Rickinson AB and Young LS (1992) Epstein–Barr virus latent gene transcription in nasopharyngeal carcinoma cells: coexpression of EBNA1, LMP1, and LMP2 transcripts. *J Virol* **66**: 2689–2697

Chan JKC, Sin VC, Wong KF, Ng CS, Tsang YW, Chan CH, Cheung MMMC and Lau WH (1997) Nonnasal lymphoma expressing the natural killer cell marker CD56: a clinicopathologic study of 49 cases of uncommon aggressive neoplasm. *Blood* **89**: 4501–4513

Cohen JL and Lekstrom K (1999) Epstein–Barr virus BARF1 protein is dispensable for B-cell transformation and inhibits alpha interferon secretion from mononuclear cells. *J Virol* **73**: 7627–7632

Deacon EM, Pallesen G, Niedobitek G, Crocker J, Brooks L, Rickinson AB and Young LS (1993) Epstein–Barr virus and Hodgkin's disease: transcriptional analysis of virus latency in the malignant cells. *J Exp Med* **177**: 339–349

Fahraeus R, Rumo L, Rhim JS and Klein G (1990) Morphological transformation of human keratinocytes expressing the LMP gene of Epstein–Barr virus. *Nature* **345**: 447–449

Hamilton-Dutoit SJ, Rea D, Raphael M, Sandvej K, Delecluse HJ, Gisselbrecht C, Marelle L, van Krieken HJ and Pallesen G (1993) Epstein–Barr virus-latent gene expression and tumour cell phenotype in acquired immunodeficiency syndrome-related non-Hodgkin's lymphoma. Correlation of lymphoma phenotype with three distinct patterns of viral latency. *Am J Pathol* **143**: 1072–1085

Harabuchi Y, Yamanaka N, Kataura A, Imai S, Kinoshita T, Mizuno F and Osato T (1990) Epstein-Barr virus in nasal T-cell lymphomas in patients with lethal midline granuloma. *Lancet* **335**: 128–130

Harris NL, Jaffe ES, Diebold J, Flandrin G, Muller-Hermelink K, Vardiman J, Lister TA and Bloomfield CD (1999) World Health Organization Classification of neoplastic diseases of the hematopoietic and lymphoid tissues: Report of the Clinical Advisory Committee Meeting – Airlie House, Virginia, November 1997. *J Clin Oncol* **17**: 3835–3849

Henderson S, Rowe M, Gregory C, Croom-Carter D, Wang F, Longnecker R, Kieff E and Rickinson A (1991) Induction of bcl-2 expression by Epstein–Barr virus latent membrane protein 1 protects infected B cells from programmed cell death. *Cell* **65**: 1107–1115

Henderson S, Huen D, Rowe M, Dawson C, Johnson G and Rickinson A (1993) Epstein–Barr virus-coded BHRF1 protein, a viral homologue of Bcl-2, protects human B cells from programmed cell death. *Proc Natl Acad Acad Sci USA* **90**: 8479–8483

Hsu DH, Malefyt RDW, Fiorentino DF, Dang MN, Vieira P, deVries J, Spits H, Mosmann TR and Moore KW (1990) Expression of Interleukin-10 activity by Epstein–Barr virus protein BCRF1. *Science* **250**: 830–832

Hudson GS, Bankier SC, Satchwell and Barrell BG (1985) The short unique region of the B95.8 Epstein–Barr virus genome. *Virology* **147**: 81–85

Iwatsuki K, Ohtsuka M, Harada H, Han G and Kaneko F (1997) Clinicopathologic manifestations of Epstein–Barr virus-associated cutaneous lymphoproliferative disorders. *Arch Dermatol* **133**: 1081–1086

Jaffe ES, Costa J, Fauci AS, Cossman J and Tsokos M (1983) Malignant lymphoma and erythrophagocytosis simulating malignant histiocytosis. *Am J Med* **75**: 741–749

Jaffe ES, Chan JKC, Su IJ, Frizzera G, Mori S, Feller AC and Ho FCS (1996) Report of the workshop on nasal and related extranodal angiocentric T/natural killer cell lymphomas. *Am J Surg Pathol* **20**: 103–111

Kerr BM, Lear AL, Rowe M, Croom-Carter D, Young LS, Rookes SM, Gallimore PH and Rickinson AB (1992) Three transcriptionally distinct forms of Epstein–Barr virus latency in somatic cells hybrids: cell phenotype dependence of viral promotoer usage. *Virology* **187**: 189–201

Levitskaya J, Coram M, Levitsky V, Imreh S, Steigerwald-Mullen PM, Klein G, Kurilla MG and Masucci MG (1995) Inhibition of antigen processing by the internal repeat region of Epstein–Barr virus nuclear antigen-1. *Nature* **375**: 685–688

Miller G (1990) The switch between latency and replication of Epstein–Barr virus. *J Infect Dis* **161**: 833–844

Miyazaki I, Cheung RK and Dosch HM (1993) Viral interleukin 10 is critical for the induction of B cell growth transformation by Epstein–Barr virus. *J Exp Med* **178**: 439–447

Moore KW, Vieira P, Fiorentino DF, Trounstine ML, Khan TA and Mosmann TR (1990) Homology of cytokine synthesis inhibitory factor (IL-10) to the Epstein–Barr virus gene BCRF1. *Science* **248**: 1230–1234

Ohtsuka M, Iwatsuki K, Kaneko R, Akiba H, Kikuchi S, Harada H and Kaneko F (1999) Epstein–Barr virus-associated lymphoid hyperplasia of the eyelid characterized by intramuscular infiltration. *Br J Dermatol* **140**: 358–359

Oudejans JJ, Jiwa M, van den Brule AJC, Grasser FA, Horstman A, Vos W, Kluin PM, van der Valk P, Walboomers JMM and Meijer CJLM (1995a) Detection of heterogeneous Epstein–Barr virus gene expression patterns within individual post-transplanation lymphoproliferative disorders. *Am J Pathol* **147**: 923–933

Oudejans JJ, van den Brule AJC, Jiwa NM, de Bruin PC, Ossenkoppele GJ, van der Valk P, Walboomers JMM and Meijer CJLM (1995b) BHRF1, the Epstein–Barr virus (EBV) homologue of the Bcl-2 proto-oncogene, is transcribed in EBV-associated B-cell lymphomas and in reactive lymphocytes. *Blood* **86**: 1893–1902

Raab-Traub N and Flynn K (1986) The structure of the termini of the Epstein–Barr virus as a marker of clonal cellular proliferation. *Cell* **47**: 883–889

Rickinson AB, Young LS and Rowe M (1987) Influence of the Epstein–Barr virus nuclear antigen EBNA2 on the growth phenotype of virus-transformed B cells. *J Virol* **61**: 1310–1317

Rowe M, Rowe DT, Gregory CD, Young LS, Farrell PJ, Rupani H and Rickinson AB (1987) Differences in B cell growth phenotype reflect novel patterns of Epstein–Barr virus latent gene expression in Burkitt's lymphoma cells. *EMBO J* **6**: 2743–2751

Sample J, Brooks L, Sample C, Young LS, Rowe M, Gregory C, Rickinson AB and Kieff E (1991) Restricted Epstein–Barr virus protein expression in Burkitt lymphoma is due to a different Epstein–Barr nuclear antigen 1 transcriptional initiation. *Proc Natl Acad Sci USA* **88**: 6343–6347

Schaefer BC, Woisetschlaeger M, Strominger JL and Speck SH (1991) Exclusive expression of Epstein–Barr virus nuclear antigen 1 in Burkitt lymphoma arises from a third promoter, distinct from the promoters used in latently infected lymphocytes. *Proc Natl Acad Sci USA* **88**: 6550–6554

Su IJ and Hsieh HC (1992) Clinicopathological spectrum of Epstein–Barr virus-associated T cell malignancies. *Leukemia Lymphoma* **7**: 47–53

Su IJ, Hsieh HC, Lin KH, Kao WCL, Chen CL, Cheng AL, Kadin ME and Chen JY (1991) Aggressive peripheral T-cell lymphoma containing Epstein–Barr virus DNA: A clinical, pathologic and molecular analysis. *Blood* **77**: 799–808

Sugiura M, Imai S, Tokunaga M, Koizumi S, Uchizawa M, Okamoto K and Osato T (1996) Transcription analysis of Epstein–Barr virus gene expression in EBV positive gastric carcinoma: unique viral latency in the tumour cells. *Br J Cancer* **74**: 625–631

Tao Q, Robertson KD, Manns A, Hildesheim A and Ambinder RF (1998) Epstein–Barr virus in endemic Burkitt's lymphoma: molecular analysis of primary tumour tissue. *Blood* **91**: 1373–1381

Wang D, Liebowitz D and Kieff E (1985) An EBV membrane protein expressed in immortalized lymphocytes transforms established rodent cells. *Cell* **43**: 831–840

Xu ZG, Iwatsuki K, Ohtsuka M, Oyama N, Matsui T and Kaneko F (2000) Polymorphism analysis of Epstein–Barr virus isolates from patients with cutaneous NK/T-cell lymphoproliferative disorders: a possible relation to the endemic occurrence of these diseases in Japan. *J Med Virol* **62**: 239–246

Young LS, Alfieri C, Hennessy K, Evans H, Ohara C, Anderson KC, Ritz J, Shapiro RS, Rockinson A and Kieff E (1989) Expression of Epstein–Barr virus transformation-associated genes in tissues of patients with EBV lymphoproliferative disease. *N Engl J Med* **321**: 1080–1085

Zeidler R, Eissner G, Meissner P, Uebel S, Tamp R, Lazis S and Hammerschmidt W (1997) Downregulation of TAP1 in B lymphocytes by cellular and Epstein–Barr virus-encoded interleukin-10. *Blood* **90**: 2390–2397

*© 2001 Cancer Research Campaign* 
